# Application of waste iron in wet flue gas desulfurization (WFGD) wastewater treatment

**DOI:** 10.1007/s11356-024-35646-z

**Published:** 2024-11-30

**Authors:** Dominika Ścieżyńska, Maciej Majewski, Susmita Nath, Dominika Bury, Agnieszka Jastrzębska, Jan Bogacki, Piotr Marcinowski

**Affiliations:** 1 Faculty of Building Services, Hydro and Environmental Engineering, Warsaw University of Technology, Ul. Nowowiejska 20 00-653, Warsaw, Poland; 2 Faculty of Mechatronics, Warsaw University of Technology, Ul. Św. Andrzeja Boboli 8, 02-525 Warsaw, Poland

**Keywords:** Flue gas desulfurization, Wastewater treatment, Zero-valent iron, Modified Fenton process

## Abstract

The wet flue gas desulfurization (WFGD) procedure results in wastewater containing a complex mixture of pollutants, including heavy metals and organic compounds, which are hardly degradable and pose significant environmental challenges. Addressing this issue, the proposed approach, incorporating waste iron shavings as a heterocatalyst within a modified Fenton process, represents a sustainable and effective solution for contaminants degrading in WFGD wastewater. Furthermore, this study aligns with the Best Available Techniques (BAT) regulations by meeting the requirement for compound oxidation—replacing the chlorine utilization with the generation of highly reactive radicals—and coagulation, which completes the treatment process. This method introduces an innovative use of waste-derived iron shavings in a BAT-compliant technology, providing a sustainable and cost-effective alternative to conventional treatments. The study focuses on process kinetics and optimization parameters, achieving approximately 48% total organic carbon (TOC) removal in 90 min at an optimal pH 3, using 1998 mgL^−1^ H_2_O_2_ under UV light. Analysis of variance revealed that the process efficiency depended more significantly on pH than time duration or the H_2_O_2_ dose. Catalyst’s characterization, including the use of microscopic techniques, including electron microscopy, laser diffraction, X-ray fluorescence, Raman spectroscopy, and UV spectroscopy, indicates its stability and great reusability with consistent TOC decrease across three process cycles. This research demonstrates a cost-effective, environmentally friendly approach to wastewater treatment, advancing sustainable methodologies through the repurposing of waste materials and underscoring the catalyst’s reuse potential.

## Introduction

Intense technological development results in a constantly increasing demand for electrical energy. Both modern economies and millions of individual households rely on energy. In the energy sector, conventional power plants produce electrical and thermal energy through fuel combustion processes and constitute a large contribution to the current energy sector. Therefore, a key challenge relates to developing improved methods and minimizing the negative impact of conventional power plants on the environment.

In conventional power plants, the chemical energy contained in fuel (coal, oil, gas, etc.) is converted into electrical energy. This process occurs in several stages. Firstly, high-temperature and high-pressure steam (steam thermal energy) is generated in boilers by fuel combustion. Then, the steam enters turbines where it expands and performs mechanical work. The obtained mechanical energy is delivered to the generator and transformed into electrical energy. As a result of combustion, flue gases containing pollutants from the burned fuel are produced. The main pollutants are carbon dioxide (CO_2_), nitrogen and sulfur oxides (NO_x_, SO_x_), hydrogen chloride and fluoride (HCl, HF), persistent organic compounds (POCs) including polycyclic aromatic hydrocarbons (PAHs), polychlorinated dioxins, furans, and biphenyls (PCDDs, PCDFs, and PCBs respectively), volatile organic compounds (VOCs), boron (B), and heavy metals, especially iron (Fe), mercury (Hg), nickel (Ni), vanadium (V), cadmium (Cd), and thallium (Tl) and their compounds, as well as total particulate matter and its fractions such as PM1, PM2.5, and PM10.

High SO_x_ removal efficiency can be achieved through several treatment methods such as dry, semi-dry, and wet flue gas desulfurization technologies. Note that among these different methods, wet flue gas desulfurization (WFGD) installation is a mature and widespread technology (Li et al. 2022). The WFDG is mainly carried out using the wet limestone-gypsum method and spraying limestone milk (Zhang et al. 2019). Power units are equipped with wet flue gas dedusting installations based on electrostatic precipitators with a dust removal efficiency even of 99.8% (Hanif et al. 2020). In the WFGD process, sulfur dioxide reacts with limestone milk to produce calcium sulfate (IV) (CaSO_3_). The associated oxidation reaction results in the formation of calcium sulfate (VI) (CaSO_4_, gypsum) (Lee et al. 2018). Moreover, apart from SO_2_, other acidic components, such as SO_3_, HCl, HF, Hg, and ash, are removed from the process. Highly polluted effluents, which are generated after gypsum precipitation and dewatering, are called WFGD wastewater (Yan et al. 2019).

The WFGD wastewater is characterized by variability in qualitative and quantitative composition, depending on the type of fuel used, unit load, and hydraulic load of the installation. The generated effluents have high values of indicators (parameters) such as total organic carbon (Toh2C), chemical oxygen demand (COD), total suspended solids (TSS), mineral oils, ammonia (NH_3_), heavy metals including antimony (Sb), arsenic (As), cobalt (Co), manganese (Mn), thallium (Tl), vanadium (V), tin (Sn), cadmium (Cd), chromium (Cr), copper (Cu), mercury (Hg), nickel (Ni), lead (Pb), zinc (Zn), adsorbable organic halogens (AOX), extractable organic halogens (EOX), PAHs (as benzo-α-pyrene, BAP), cyanides (CN), sulfides (S^2−^), sulfates (IV) (SO_3_^2−^), sulfates (VI) (SO_4_^2−^), total nitrogen (TN), total phosphorus (TP), fluorides (F^−^), phenols, PCDDs, and PCDFs (as toxicity equivalent, TEQ). Another compound that drastically increases salinity is chlorides mainly from WFGD (Liu et al. 2023). Moreover, a variety of organic compounds, including hydrocarbons, esters, siloxanes, halogenated hydrocarbons, and more, can be found in WFGD wastewater (Liu et al. 2022).

Furthermore, desulfurization processes often require the use of alkaline reagents, which results in high pH of WFGD wastewater. Depending on the processes used, the temperature of the effluents ranges from 40 to 70 °C. WFGD wastewater often has a yellow or brown color, caused by the presence of dissolved organic compounds and heavy metals. Moreover, the wastewater parameters are influenced by the quality of raw water, the desulfurization process applied, and even the treatment plant configuration. Many of the mentioned substances are persistent compounds, toxic to aquatic organisms, and harmful to the environment. These are mainly inorganic pollutants. The concentration of organic compounds in effluents is relatively small. Untreated effluents can be a significant source of environmental pollution. Toxic substances can accumulate in the food chain and cause chronic diseases and developmental disorders in humans and wildlife. These compounds may also exhibit carcinogenic properties and cause respiratory failure and death.

Effluent treatment is essential to meet legal requirements and protect the environment from pollution. European Union environmental laws, particularly the IPPC Directive (Integrated Pollution Prevention and Control), promotes the use of Best Available Techniques (BAT), which outline industry-specific standards and best practices for minimizing industrial environmental impacts. To meet strict emission standards, consistent with BAT conclusions, two-stage precipitation processes are commonly used to treat WFGD wastewater. Initially, WFGD wastewater is oxidized with sodium hypochlorite (NaOCl). Then, gypsum is separated by raising the pH using calcium hydroxide, Ca(OH)_2_, and iron (III) chloride, FeCl_3_ for coagulation/flocculation. The sludge from this stage can be partially recycled. In the second stage, metals are precipitated by adding organic sulfides. The sludge generated in this stage must be removed. The sludge from WFGD wastewater treatment, as well as the previously separated gypsum, are treated as coal combustion residuals (CCRs). This group also includes fly ash and bottom ash (Senior et al. 2020). Gypsum and some fractions of ash are utilized as commercial products in the construction industry. However, the remaining materials classified as CCRs are deposited in landfills and can impact the environment. The assessment of the impact of such materials relies on leachability tests and measurements of indicators in groundwater and surface water (Deonarine et al. 2023).

The composition of WFGD wastewater and the content of trace elements in wastewater and residues after coal combustion depend on their form during the combustion process and their volatility. This is related to the combustion process during which they are released from coal. Some of them may undergo re-condensation in the flue gas cleaning process and get into the wastewater, while others may undergo condensation and/or adsorption on suspended ash (Senior et al. 2020).

It should be noted that the rigor regarding the parameters of discharged wastewater is constantly increasing. This means that soon, the currently used methods of wastewater treatment in conventional power plants will become insufficient. Wastewater from combustion gases desulfurization is treated by physicochemical methods (chemical precipitation, coagulation, electrocoagulation, electrochemical processes, adsorption, ion exchange), biological methods, membrane processes (membrane distillation, microfiltration, ultrafiltration, nanofiltration, reverse osmosis), and evaporation (spray drying, mechanical vapor compression, thermal vapor compression) (Gingerich et al. 2018).

Reverse osmosis, microfiltration, and ultrafiltration, or ion exchange, are very effective methods of treatment. However, they have certain drawbacks, mainly related to high maintenance costs and energy consumption (Shin et al. 2022). Therefore, further research is necessary to find effective, yet inexpensive and applicable methods, or to upgrade wastewater treatment.

Anderson et al. recovered 89% of water from FGD wastewater and achieved a reduction of all tested ions (Na^+^, K^+^, Ca^2+^, Mg^2+^, B, Cl^−^, SO_4_^2−^) by 99.7% in a process coupling osmosis with membrane distillation, using a flat, asymmetric membrane made of cellulose triacetate in the osmosis process and a thin active layer of polytetrafluoroethylene deposited on a layer of polypropylene in membrane distillation (Anderson et al. 2021). The required removal of some salinity-causing substances before using membrane distillation can be accomplished by the chemical precipitation of Ca^2+^ and Mg^2+^ (a significant share of these cations in the total of all ions in the solution) using Na_2_CO_3_ and pH correction, supplemented with the addition of polyacrylamide flocculant (Li et al. 2021).

Despite its high effectiveness, the hybrid process has its drawbacks. One of the major ones is membrane fouling in microosmosis, resulting in a significant decrease in the overall effect efficiency and the need to replace the membrane itself (Lee et al. 2018; Li et al. 2021). Tang et al. aimed to reduce the negative impact on the membrane in the ultrafiltration process in a hybrid system by applying chemical precipitation-UF, determining the optimal process conditions. They achieved a 98% reduction in hardness using CaO and Na_2_CO_3_ precipitation in proportions of [CaO]/[Mg^2+^] and [Na_2_CO_3_]/[Ca^2+^] at 2.5 and 2.7 respectively. The chemical precipitation process was conducted without the addition of a flocculant (Tang et al. 2023). Despite the high efficiency, the use of membranes is economically unjustified due to the process drawbacks associated with treating the large volumes of wastewater generated in the desulfurization process.

The high chloride ion content in wastewater limits the possibility of using biological methods in wastewater treatment due to its high ionic strength. Cui et al. proposed the use of an electrochemical process, in which oxidation to chlorine (Cl_2_) occurs at the anode (pure Ti electrode), and hydrogen (fuel) is evolved at the cathode (Ru, Ir-coated Ti electrode with a Ti dopant). Ions migrate in the electrodialysis process. The results prove that the electrolytic–electrodialysis process for WFGD treatment can achieve WFGD zero discharge and resource utilization, and be economically feasible (Cui et al. 2017).

Li et al. proposed a method for removing chloride ions from WFGD wastewater through electrocoagulation using two-layer structures (Li et al. 2019). Wastes generated during coal combustion can also be successfully used to remove Cl^−^ from WFGD wastewater. Volatile ashes after alkaline roasting obtain a porous hydrotalcite structure on their surface. It is possible to reduce even by 68.1% the content of Cl^−^ under optimal conditions, i.e., using an adsorbent dose of 10 g/L, at pH 8, at a temperature of 60 °C (Qi et al. 2020). Removal of Cl^−^ from FGD wastewater with an efficiency of 48.1–60.3% can also be achieved by precipitation as Friedel’s salt (Ca_4_Al_2_(OH)_12_Cl_2_). The effectiveness of the process depends on the molar ratios of SO_4_^2−^ (concentration in wastewater), Cl^−^ (concentration in wastewater), Ca^2+^ (amount of CaO dosed during gypsum precipitation and precipitation of Friedel’s salt), and Al^3+^ (NaAlO_2_ dosed at the stage of Friedel’s salt precipitation) (Xin et al. 2020; Ye et al. 2021; Liu et al. 2023).

The desalination process can be preceded by a pre-treatment process, during which organic compounds resistant to biodegradation are decomposed. Shin et al. conducted tests on synthetic WFGD wastewater, containing cimetidine, bisphenol-A, benzoic acid, and sulfamethoxazole as substances resistant to biodegradation. The wastewater was subjected to photochemical oxidation using a TiO_2_ nanotube system. W-UV-A lamps (300–400 nm) with a maximum radiation at a wavelength of 352 nm were used as a light source. The pre-treated wastewater was subjected to demineralization using ion exchange membrane systems, mounted perpendicular to the flow direction of the treated wastewater and parallel to the electrodes inducing ion migration. The use of such a system allowed for the effective decomposition of organic pollutants, although the overall effect of the process decreases with the increasing salinity of raw WFGD wastewater (Shin et al. 2022).

Biological methods in WFGD wastewater treatment are applied after pre-treatment, which aims to reduce salinity and remove toxic substances such as organic compounds resistant to biodegradation or heavy metals. Yan et al. investigated the possibilities of reducing sulfates using sulfate-reducing bacteria from BY7, SR10, and SR3 strains after prior chemical precipitation. The application of such a low-cost hybrid process allowed for a 33% reduction in total organic carbon (TOC) value, a 97% reduction in suspended solids, and a 97–100% removal of heavy metals (Yan et al. 2019, 2022). Even up to 14 substances (depending on conditions) resistant to biodegradation, out of 25 detected in raw wastewater, were completely removed (Yan et al. 2019).

Zhang et al. investigated the effect of Pb and Hg content in WFGD wastewater on sulfate-reducing bacteria. A reduction in sulfate content by 72.5%, a decrease in COD by 86.4%, and over 99.5% removal of Pb and Hg were achieved. Most heavy metals were precipitated as sulfides. Only 20% of Pb(II) and 1.8% of Hg(II) were biosorbed onto activated sludge flocs and bound to organic compounds. It was also found that the applied biological reactor can successfully operate for a longer period, and the impact of heavy metals at the concentration determined in FGD wastewater on the biota is negligible (Zhang et al. 2016).

Taking the above into consideration, this study aimed to adopt an effective treatment method in response to continuously tightening emission standards, including BAT criteria. The WFGD wastewater are characterized by high-chloride content, which can result in chlorination, which in turn may lead to the formation of toxic chlorinated organic compounds. This challenge has encouraged the search for alternative oxidants. The Fenton process was proposed since it is a chemical oxidation method involving the generation of highly reactive hydroxyl radicals (HO^•^) through the reaction between hydrogen peroxide (H_2_O_2_) and ferrous iron (Fe^2+^), according to Eq. (1).

1 $${\text{Fe}}^{2+}+{\text{H}}_{2}{\text{O}}_{2}\to {\text{Fe}}^{3+}+{\text{OH}}^{-}+{\text{HO}}^{\cdot }$$

Hydroxyl radicals generated are highly reactive and can oxidize organic pollutants present in the wastewater, breaking them down into smaller, less harmful molecules through processes such as hydrogen abstraction, addition, and decomposition. In the traditional Fenton process, iron ions are introduced as an acidified salt, which increases the salt content in wastewater, creating additional environmental challenge. Therefore, proposed modified heterogeneous process uses metallic iron. However, to ensure the process is effective, iron must undergo surface corrosion which occurs under low-pH conditions.

In the heterogeneous Fenton process, a solid catalyst provides a surface for the adsorption of organic compounds, allowing their oxidation at the near-surface sites in which HO^•^ are most efficiently generated. This enhances the efficiency of the Fenton reaction and allows for the degradation of organic pollutants at lower concentrations of hydrogen peroxide. Moreover, the final Fenton process step is coagulation—sample alkalinization conducted to remove iron from the solution, decompose the peroxide, and oxidize Fe^2+^ to Fe^3+^. What is more, at low pH conditions, iron ions remain soluble, so increasing pH stops the solubility process.

The use of solid catalysts in the heterogeneous Fenton process offers several advantages, including improved stability and reusability of the catalyst, enhanced mass transfer of reactants, and increased resistance to the inhibition effects of organic compounds present in the wastewater. In other words, the heterogeneous Fenton process represents an innovative approach to wastewater treatment that combines the principles of the Fenton reaction with the advantages of solid catalysts, offering a promising solution for the removal of organic pollutants from wastewater streams.

The use of a heterogeneous Fenton process utilizing waste iron, hydrogen peroxide, and supported by ultraviolet (UV) radiation to generate highly reactive HO^•^ seems like an ideal solution. In addition, in the presence of UV light, the H_2_O molecule photolysis is induced, and the subsequent photoreduction of Fe^3+^ ions to Fe^2+^ with the generation of HO^•^ occurs (Eq. 2).

2 $${\text{Fe}}^{3+}+{\text{H}}_{2}\text{O}-\text{hv}\to {\text{Fe}}^{2+}{+\text{HO}}^{\cdot }+{\text{H}}^{+}$$

Such action allows for reduction of the dose of active catalyst in the process.

The Fenton process and its modifications have previously been demonstrated as effective wastewater treatment methods across various industries, including heavy metals. Proposed technologies should be technically feasible and easily implementable, which these processes are.

This study introduces a novel approach to WFGD wastewater treatment, leveraging waste iron shavings as a catalyst to effectively degrade contaminants. Unlike traditional methods, this approach eliminates the use of chlorine, mitigating the risk of chloroorganic compound formation. Additionally, the utilization of highly reactive radicals generated in the UV-assisted pseudo-Fenton process enhances pollutant degradation efficiency, representing a significant advancement in wastewater treatment technology. Additionally, the redox potential of HO^•^ generated in the UV-assisted pseudo-Fenton process is superior to chlorine redox potential, reinforcing both the thorough degradation of pollutants and the unwavering adherence to regulatory standards set by Best Available Techniques (BAT). This study thus underscores its pivotal role in advancing the overarching goal of promoting environmentally responsible and sustainable practices in wastewater management.

## Materials and methods

### FGD wastewater collection

The tested WFGD wastewater samples were collected from a conventional power plant located in Poland. Wastewater was collected after gypsum separation. Samples were taken directly at the discharge. Then, they were stored at 4 °C until analysis. The samples were transparent with a yellowish color and had no odor.

### Catalyst characterization

Zero valent iron (ZVI, Fe^0^) has already been proven to be an effective material for the treatment of large amounts of organic pollutants that can be found in the environment (Bogacki et al 2018). This is due to its catalytic properties, low toxicity, and relatively low cost. Moreover, it has magnetic properties which allow for an easy separation from the solution. Furthermore, to make ZVI utilization even more efficient, environmentally friendly, and affordable, in this work, metallic iron was collected as a waste material from machining processes.

The first step of waste iron characterization included the material structure and morphology analysis. To prove its effectiveness and precisely examine the structure of the waste iron grains, a DMS1000 microscope, and OPTA-TECH MB 200 (OPTA-TECH, Warsaw, Poland) microscope with additional light source was used for analysis, resulting in high-quality digital images. To obtain more detailed information on the material’s surface, a scanning electron microscope Hitachi S3500N (SEM) from Japan was used. SEM analysis was performed under precise conditions, including an accelerating voltage of 15 eV, which made it possible to obtain reliable and accurate results. To improve conductivity, the samples’ surface was sputtered with gold using a BAL-TEC SCD 005. The grain size was carefully analyzed by using the Mastersizer (Malvern Mastersizer 2000 MU, Malvern, UK). To obtain more detailed information and mapping, a scanning electron microscope Hitachi S3500N (SEM) (Japan) was used to perform the analysis, including an accelerating voltage of 15 eV, which made it possible to obtain reliable and accurate results. To improve conductivity, the samples’ surface was sputtered with gold using a BAL-TEC SCD 005.

The X-ray fluorescence (XRF) technique, using a PI 100 spectrometer from Polon-Izot, Warsaw, Poland, equipped with a rhodium (Rh) anode was applied to confirm the purity of materials and the presence of iron in the samples. The analysis was registered using a drift detector (SSD) with a resolution of 125–140 eV and a multilayer monochromator, which made it possible to precisely determine the samples’ chemical composition. Moreover, the waste iron chemical structure was presented as the bonding and vibrations in the sample studied by Raman spectroscopy (Renishaw InVia confocal Raman microspectrometer).

The waste iron surface oxidation was verified by the direct band gap presence analysis. The band gap was evaluated using the Tauc plot method based on the absorbance measurements diffused reflectance (DRS); dual-beam scanning was employed using a UV–visible spectrophotometer (Evolution 220, ThermoFisher Scientific, Waltham, MA, USA) with an integrating sphere. Measurements were carried out in the wavelength range 220–1100 nm, with an integration time of 0.3 s, a resolution of 1 nm, and a scanning speed of 200 nm/min.

A comprehensive examination of porosity in waste iron offers invaluable insights into both its internal structure and surface properties. These aspects play pivotal roles in determining the material’s reactivity, surface area, or ability to adsorb pollutants. Moreover, such a study provides a complete picture of the waste iron characteristics, which is important for assessing its suitability for various applications.

### Wastewater treatment process

#### UV/Fe^0^/H_2_O_2_

Wastewater was treated using the UV/Fe^0^/H_2_O_2_ process. The experiments were conducted in 1.5 L reactors with a 1 L sample scale. To ensure optimal reaction conditions, different doses of waste iron and 30% hydrogen peroxide (Stanlab, Lublin, Poland) were used. Various doses of the waste iron were applied: 1, 2, and 4 gL^−1^, along with different hydrogen peroxide doses in H_2_O_2_/COD ratios: 1:1, 2:1, 4:1, 8:1 to 16:1 which were 250, 500, 1000, 2000, and 4000 mgL^−1^ respectively. The initial pH of the wastewater was adjusted to 3.0 using sulfuric (VI) acid.

The wastewater samples with added reagents were stirred using a magnetic stirrer at 300 rpm (Heidolph MR3000, Schwabach, Germany). This allowed the effectively maintained suspension of catalysts throughout the sample volume, enhancing their contact with the studied material. The centrifugal force generated was sufficiently strong to detach the filings from the propeller and effectively disperse them in the sample volume.

Subsequently, the treated wastewater was exposed to medium-pressure Fe/Co 400 W lamps, type HPA 400/30 SDC, with a UVA power of 94 W (Philips, Amsterdam, Netherlands), centrally positioned 1 m above the samples. After specific exposure times (10, 30, 60, and 90 min), around 100 mL of samples was collected, and processes were stopped by raising the pH to 9.0, using a 3M NaOH solution (POCh, Gliwice, Poland). Samples were left overnight to allow hydrogen peroxide decomposition, coagulation with iron (III) hydroxide, and sedimentation.

The assessment of process efficiency involved determining the total organic carbon (TOC) using a TOC-L analyzer (Shimadzu, Kyoto, Japan) with an OCT-L8-port sampler (Shimadzu, Kyoto, Japan).

#### Reaction kinetics

In this study, the kinetics of WFGD wastewater TOC degradation were determined. To analyze the treatment processes kinetics in the experiments, different order kinetics equations and their modifications were used (Eqs. 3–6), according to previous work (Marcinowski et al. 2020):

3 $$\text{TOC}={TOC}_{0}+{e}^{-kt}$$

4 $$\text{TOC}=\left(\text{kt}+1/{\text{TOC}}_{0}\right)$$

5 $$\text{TOC}=\left({\text{TOC}}_{0}-b\right)+{e}^{-kt}+b$$

TOC = (kt + (TOC_0_ − b)^−1^)^−1^ + b

6 $$\text{TOC}={\left(kt+{\left({\text{TOC}}_{0}-b\right)}^{-1}\right)}^{-1}+b$$

where:

The “k” parameter in the equations corresponds to the reaction rate constant. It is the coefficient of proportionality. This parameter in the equations is constant.

The “b” parameter introduces a “limit” to which the results tend in the equation. In this way non-oxidizable substance process correction takes place. This parameter assumes the residual concentration, which in this case means the amount of unremoved TOC.

The “k” and “b” parameters were introduced to eliminate errors due to the deficiency of reactants or oxidants, or the presence of substances that do not undergo highly efficient oxidation reactions.

Classical kinetic models are typically based on the assumption that chemical reactions proceed until one of the reactants is fully consumed. However, in the case of complex multi-element matrices, like WFGD wastewater, various components can interact in intricate ways, causing inhibition of other ongoing reactions. Traditional kinetics models may not accurately capture the process dynamics. Hence, modified first and second-order kinetics models have been developed. The modification addresses the high-efficiency oxidation process complexity. The process consists of oxidation and secondary coagulation after the quenching of the process. This approach provides a better adjustment of the kinetic equation to actual data.

#### ANOVA

Analysis of variance—ANOVA—is a linear modeling method for evaluating relationships between variables. In the industrial wastewater treatment processes, ANOVA is used to compare the influence of various factors, such as temperature, substrate, and catalyst concentration or solvent type, on reaction kinetics. This provides a way to determine which factors have a significant effect on the reaction, which is important both for understanding the reaction mechanism and for optimizing chemical processes. Therefore, to investigate the variability of average TOC concentrations, an ANOVA with a 0.05 significance level and the model’s prediction quality greater than 0.1 was applied. These parameters allowed a reliable predictive relationship between the predicted variable and the input variable.

#### Waste iron reusability

Additionally, a waste iron reusability test was conducted without any regeneration. After concluding the process, the catalyst was separated magnetically, and a new treatment cycle was performed with a fresh wastewater sample, maintaining the same initial parameters (pH, H_2_O_2_ dose, and process time).

## Results and discussion

### WFGD wastewater characterization

The WFDG wastewater, generated in wet flue gas desulfurization, is a specific group characterized by a combination of heavy metals, suspended solids (mainly gypsum), and large amounts of various forms of nitrogenous compounds. They are furthermore loaded with a high load of organic substances. The selected wastewater samples after the gypsum separation were characterized by the standard parameter values process as in the reported work and were as follows in Table 1.

**Table 1:** Wastewater parameters

Parameter	Result
COD	250 mgL^−1^
TOC	101.2 mgL^−1^
TSS	13.0 mgL^−1^
Phenol index	0,004 mgL^−1^
N_NH4_	36 mgL^−1^
Cl^−^	16 gdm^−3^
Mineral oil index	0.11 mgL^−1^
pH	8.8

### Waste iron characterization

Research on using waste iron as a heterocatalyst has gained attention in recent years due to its potential for environmental treatment. Importantly, it is more economically feasible and environmentally friendly than typical commercial iron products.

**Fig. 1:**
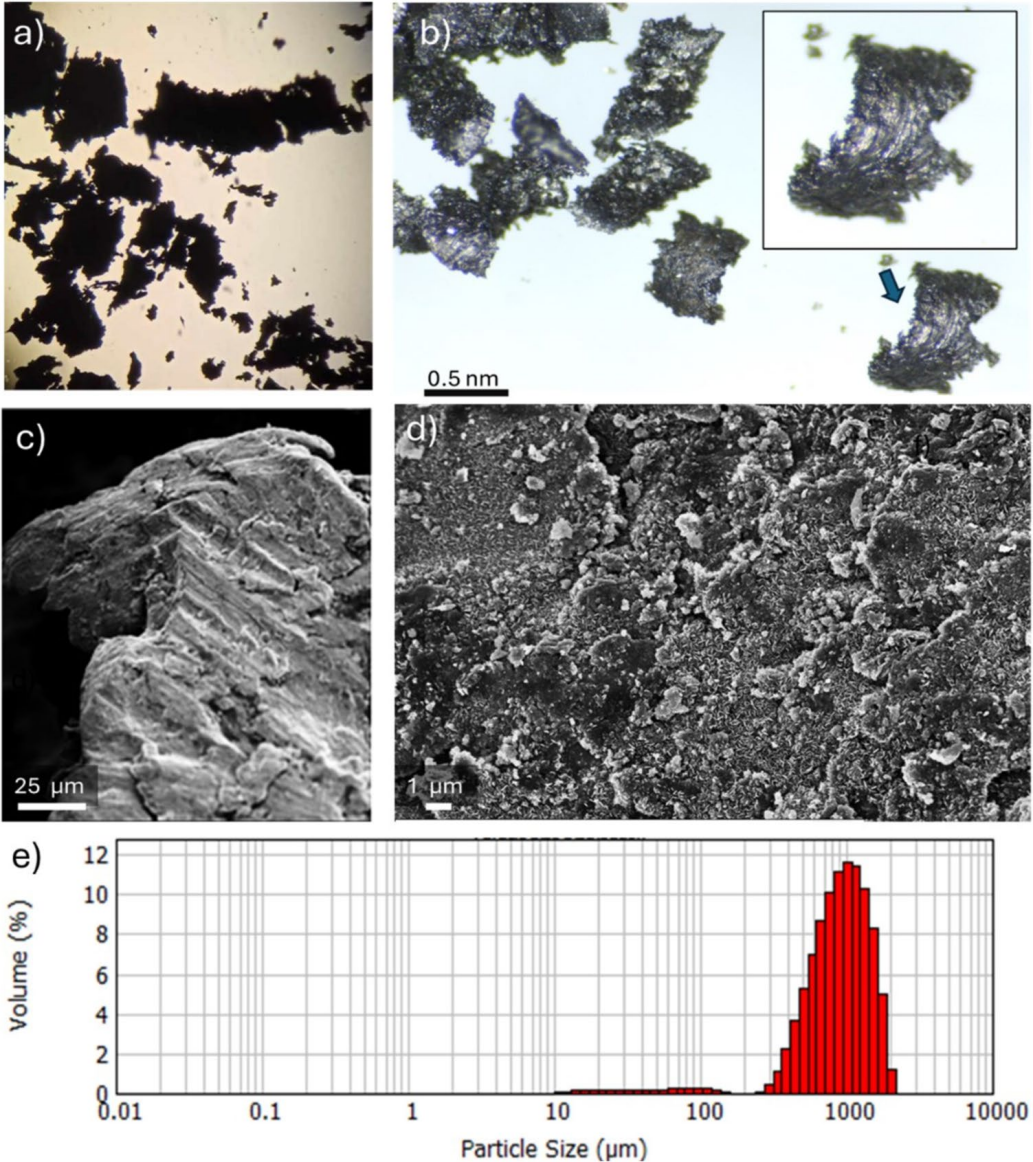
Waste iron structure and morphology: **a**, **b** microscope images, **c**, **d** SEM images, **e** grains size analysis

The waste iron used in this research is characterized by oblong shapes reminiscent of black chips with clearly visible shiny crystals (shown in Fig. 1a,b). The material structure and morphology analysis using SEM images (Fig. 1c,d) revealed waste iron grains with sharp edges as well as irregular and smooth surfaces covered in oxides, indicating oxidation of the material. The grain size was analyzed by Mastersizer (Fig. 1e**)**. The analysis showed particle sizes in the range of 500–1200 μm with the most in the 1000 μm size.

The results of XRF analysis indicated a predominant presence of iron in the catalyst structure, confirming the high purity of the material. The obtained results were devoid of additional compounds, confirming material uniformity. This is significant, as pure iron can contribute to the effective progression of catalysis.

**Fig. 2:**
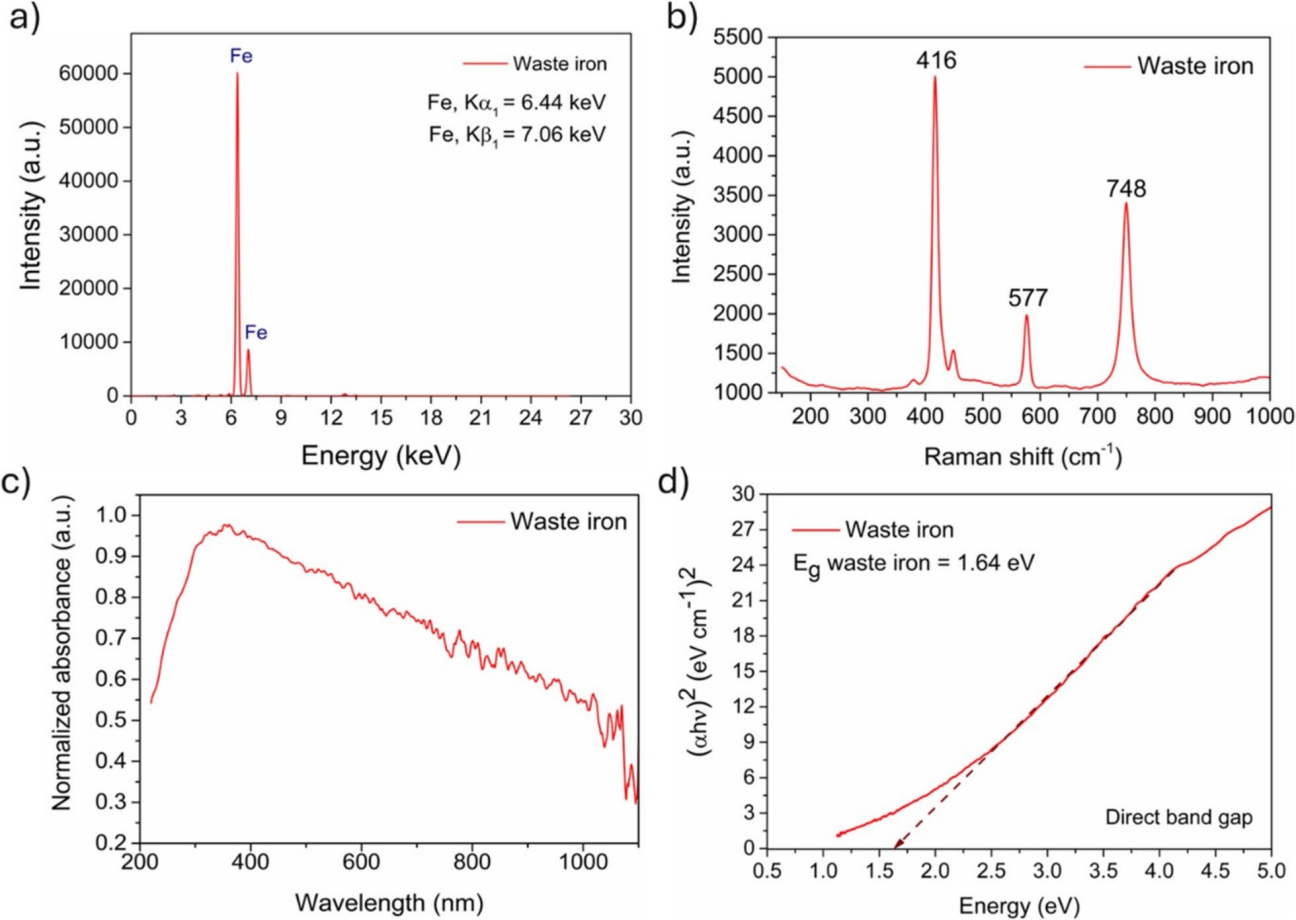
Waste iron. **a** XRF analysis, **b** Raman analysis, **c** absorbance, **d** direct band gap

The next characterization step included Raman analysis, allowing the identification of oxides on the material surface **(**Fig. 2b). The obtained results showed the presence of peaks at 416 and 577 cm^−1^, characteristic of α-Fe_2_O_3_ hexagonal plates. Simultaneously, the peak at 748 cm^−1^ indicated the presence of Fe_3_O_4_ polyhedral particles in waste iron. Similar results for magnetite and hematite were obtained by Lu and Tsai (2014) and Jesús Ruíz-Baltazar et al. (2017).

To characterize the catalyst, a detailed analysis of the optical properties of waste iron was conducted, with an emphasis on absorbance (Fig. 2c) and the direct bandgap (Fig. 2d). The absorbance spectrum results showed a clear edge near 300 nm, resulting from an internal absorption band associated with bandgap transition. The results were obtained to evaluate the bandgap of the materials, indicating the presence of oxides and the semiconductor character of the materials. Extrapolation of the bandgap value at 1.64 eV is slightly smaller than known iron varieties, especially hematite and magnetite (2.1–2.46 eV) (Millaty Abadiah et al. 2019, Ren et al. 2017), confirming a favorable band gap for UV absorption. The lower value of the band gap could be the result of combining different kind of iron in the material.

The analysis constitutes a crucial stage in identifying the chemical composition of the catalyst. In the presented analysis combinations, the predominant presence of iron was confirmed, aligning with expectations given the material’s characteristics. With over 70% iron content, it suggests that the catalyst is primarily composed of this element, which may hold significant implications for its catalytic properties.

Furthermore, the observed minimal number of additives indicates a low degree of contamination. Based on the results, it can be claimed that the waste iron is distinguished by its unique structure, exhibits high purity (Fig. 2a), and has favorable optical properties (Fig. 2c,d). These characteristics make the catalyst promising for applications, especially in the context of environmental and water treatment technologies. Its promising effective participation in photocatalysis processes is an important factor, suggesting the potential benefits of this material in the field of environmental protection.

### Wastewater treatment process

#### UV/Fe^0^/H_2_O_2_

##### Optimization process

The wastewater samples were subjected to the process with UV irradiation. The initial study step involved treatment process parameters adjusting to increase its efficiency and reduce the consumption of reagents (Dong et al. 2022; Adachi et al. 2022). The organic content was evaluated based on the total organic carbon content. The content of total organic carbon in the raw sample was 101.2 mgL^−1^. The highest decrease in the total organic carbon concentration was observed after the 90 min process in a sample with a 1 g/L Fe^0^, 999 mgL^−1^ H_2_O_2_ dosage in pH 3. The final TOC concentration was 52.69 mgL^−1^, which means a decrease of 48.51 mgL^−1^, that is, a 48% organic pollutant removal. The doses used in this process were considered optimal.

##### Effect of reagent doses

**Fig. 3:**
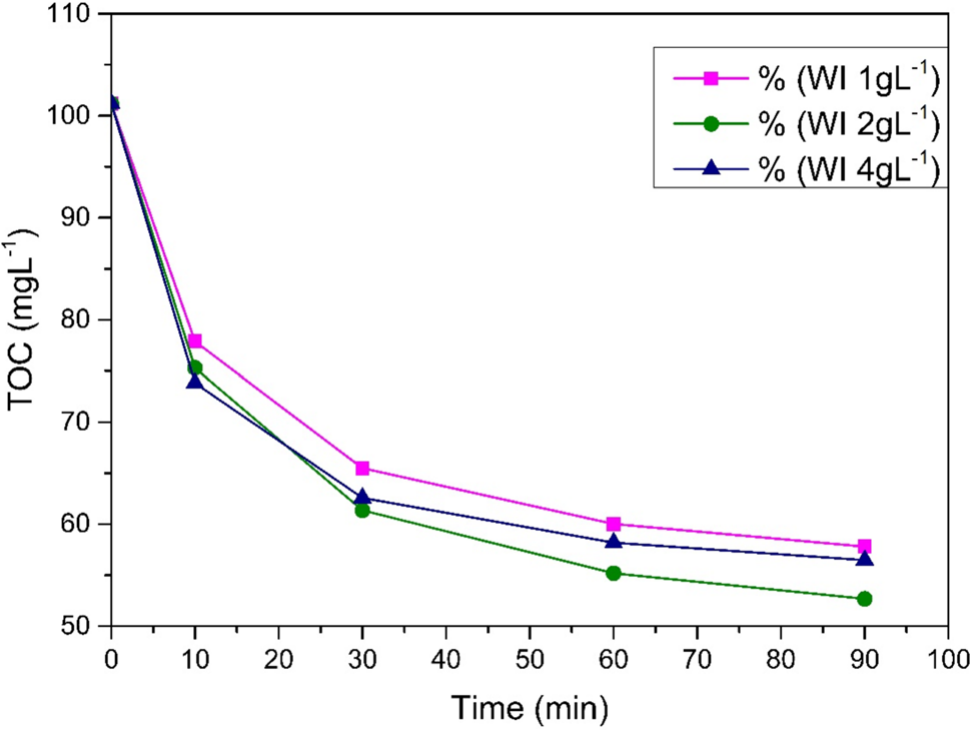
Waste iron dosage comparison, pH 3, H_2_O_2_ 2000 mgL^−1^

In the 4 gL^−1^ Fe^0^, 500 mgL^−1^ H_2_O_2_ sample case, at pH 3, the smallest difference in TOC concentration, which was 72.1 mgL^−1^, before and after the process was recorded. This corresponds to a decrease of slightly less than 29% from the initial concentration. The best results of TOC decrease were achieved using a waste iron dosage of 1 gL^−1^ (Fig. 3). Processes with various doses of catalyst: 0.5, 1.0, 2.0 g, and various doses of hydrogen peroxide: 0.75, 1.5, 3.0, and 6.0 mgL^−1^, were carried out. Both smaller and larger doses of catalyst had a negative effect on process efficiency. Augmentation of the catalyst amount increased dissolved Fe^2+^ ions, causing turbidity of the reaction mixture, thus reducing the availability of UV radiation, and leading to undesired reactions and radical scavenging (Saeed et al. 2022; Gupta et al. 2024).

##### Effect of H_2_O_2_ doses

**Fig. 4:**
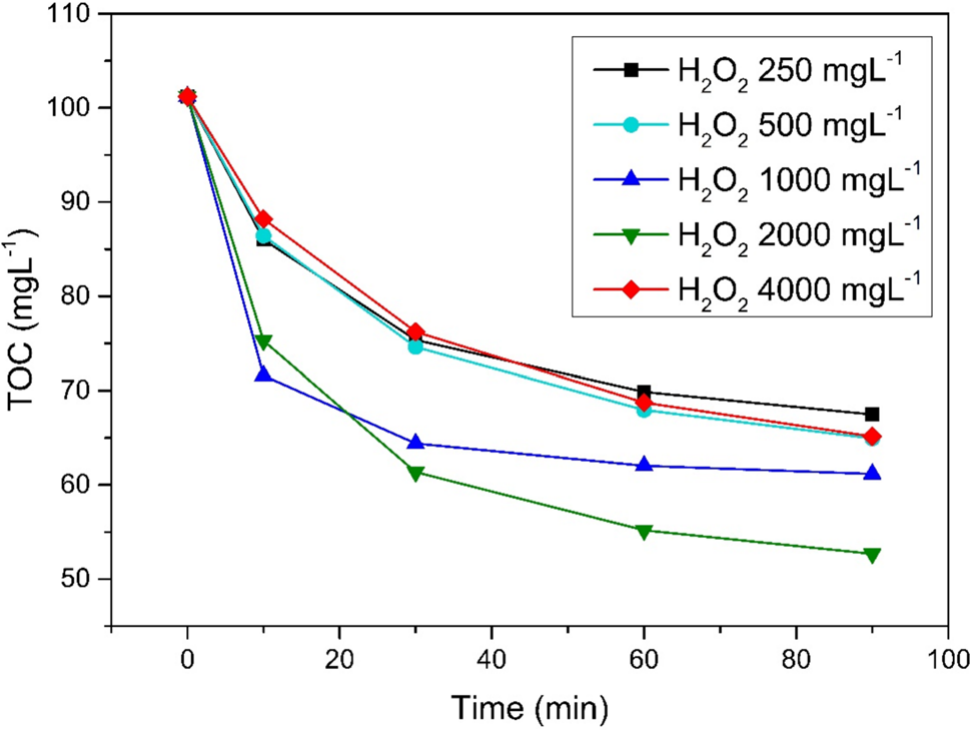
Hydrogen peroxide dosage comparison UV/Fe^0^/H_2_O_2_, pH 3, Fe^0^ 2gL^−1^

The optimal H_2_O_2_ dose was 1998 mgL^−1^ (Fig. 4). At smaller doses, rapid consumption of H_2_O_2_ was observed, which led to substrate depletion, inhibiting the decomposition process. Increased doses of hydrogen peroxide, on the other hand, generated an inhibitory effect, promoting undesirable inter-radical reactions (mutual radical quenching) and the formation of radicals with reduced oxidizing potential (Vorontsov 2019, Wang et al. 2020). Excessive amounts of H_2_O_2_ could also lead to the formation of by-products, such as iron oxides, accumulating on the surface of the catalyst and reducing the efficiency of the process over time.

Bogacki et al. (2017) treated WFGD wastewater through sedimentation, coagulation, and the Fe^0^/H_2_O_2_ process, using Hepure Ferox Target as the commercially sourced iron. The Fe^0^/H_2_O_2_ process was the most effective one, allowing to decrease COD from 394 to 204 mgL^−1^ (48.3% removal) for 15 min process time and Fe^0^/H_2_O_2_ doses 250/600 mgL^−1^. Bogacki et al. (2018) compared also homogeneous and heterogeneous Fenton process efficiency, for the treatment of FGD wastewater. The homogeneous process was a bit more effective in terms of COD removal and could be an alternative for FGD wastewater treatment BAT guidelines.

However, it should be clearly noted that FGD wastewater contains a significant amount of reduced inorganic compounds, which significantly affect COD. Their oxidation during the process will result in a significant decrease in COD, which, however, does not translate into the removal of TOC. This presumably corresponds to the inorganic compounds oxidation, such as sulfides, sulfates (IV), etc. Therefore, the TOC decrease is much smaller than the COD one. For an effective treatment process, it is crucial to maintain an appropriate reaction environment (Wang et al. 2020).

##### pH influence

**Fig. 5:**
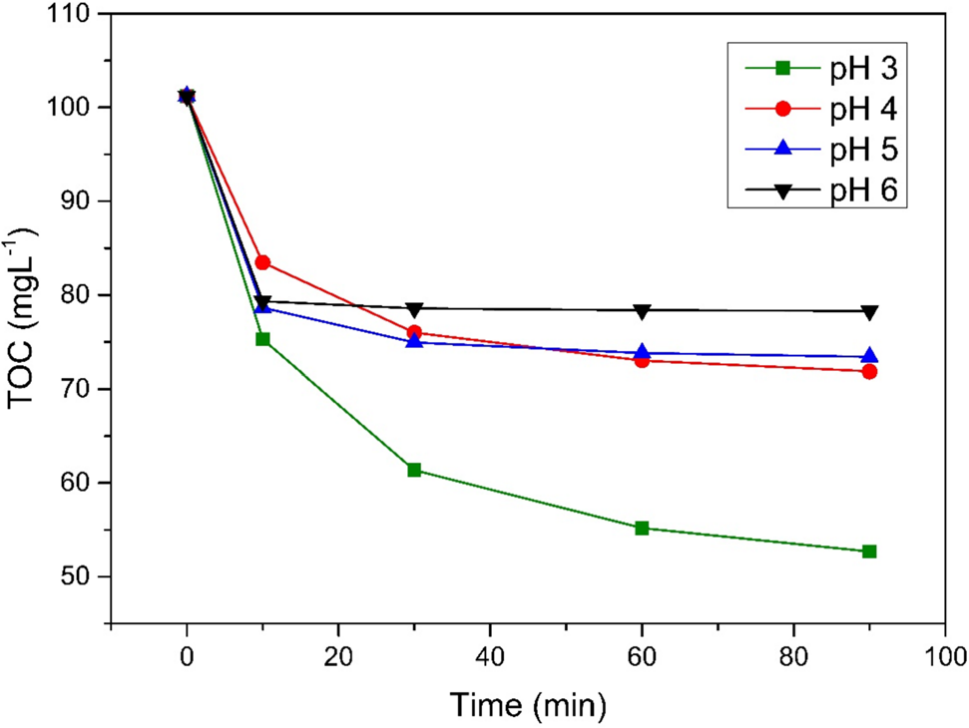
UV/Fe^0^/H_2_O_2_ processes comparison in different pH, Fe^0^ 2 gL^−1^, H_2_O_2_ 1998 mgL^−1^

The wastewater treatment efficiency decreased progressively with rising pH levels (Fig. 5).

The process efficiency, assessed by the decrease in TOC values in the FGD wastewater treatment process, dropped from 52 to 22.61% as the pH of the solution increased. Higher pH values exert a greater impact on the equilibrium of photoactive iron compounds. At pH 3, Fe^3+^ and Fe(OH)_2_^+^ and nearly evenly distributed (Gomathi Devi et al. 2011). Both low and high pH levels disrupt the equilibrium of photoactive forms, thereby reducing process efficiency. Additionally, higher pH triggers the coagulation of Fe^3+^ ions while hydrogen peroxide dissociates more readily into water and oxygen. Consequently, fewer radicals are generated. (Kremer 2021). Lowering the pH to 3 promoted the HO^•^ potential, which in turn increased the efficiency of the process. An increase in pH above 4.0 led to a drastic reduction in catalytic activity, mainly due to the insolubility of Fe^3+^ ions and the formation of a corrosion layer on the catalyst surface.

### Reaction kinetics

Chemical kinetics refers to the rate of chemical reactions study and the factors that affect it. Kinetics studies analyze how fast a chemical reaction occurs and how various factors (substrate concentration, temperature, pressure, type of solvent, or the presence of a catalyst) affect this rate and the course of the reaction itself (Bury et al. 2023). Knowing process kinetics provides an opportunity to design more efficient and environmentally sustainable systems. In addition, kinetic research provides the basis for new technologies, materials, and substance development, stimulating innovation and economic growth.

**Fig. 6:**
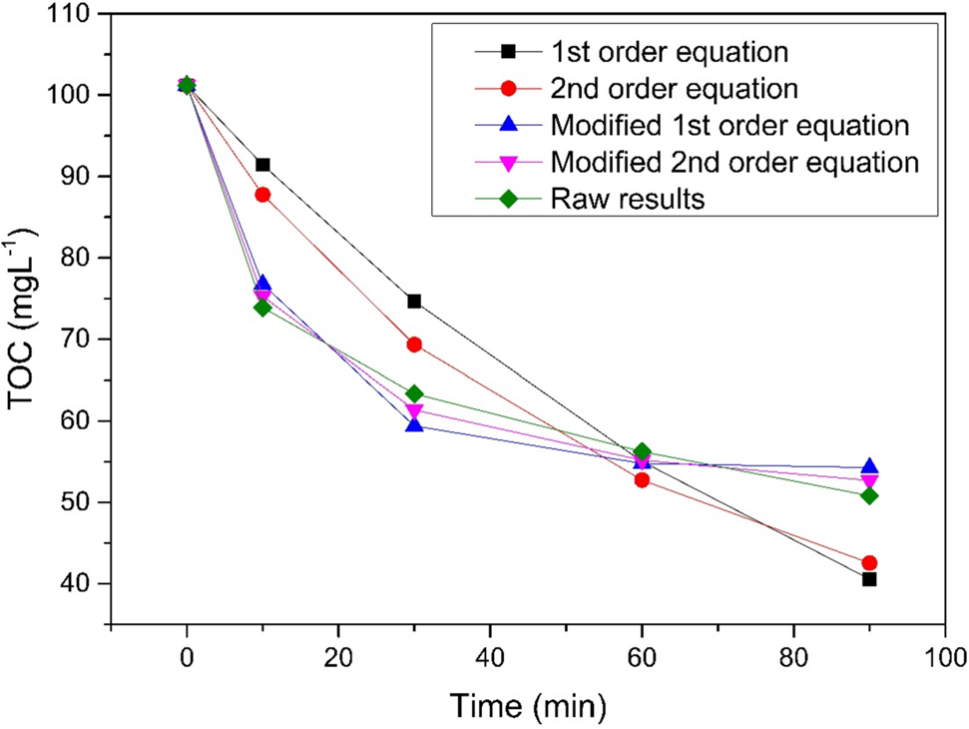
UV/Fe^0^/H_2_O_2_ kinetic calculations comparison, pH 3, Fe^0^ 1gL^−1^, H_2_O_2_ 1998 mgL^−^.^1^

The implementation of second-order equation modifications, with a rate constant *k* of 1.67 × 10⁻^3^, best represents the actual progression of the wastewater treatment reaction. The optimal duration of the process was set at 90 min. A prolonged process running did not result in any increase in process efficiency. The fastest reaction occurred in the first 30 min, after which the process slowed down noticeably, and the observed reduction in TOC values was no longer significant. The relationship between actual results and kinetic calculations is shown in Fig. 6.

### ANOVA

**Fig. 7:**
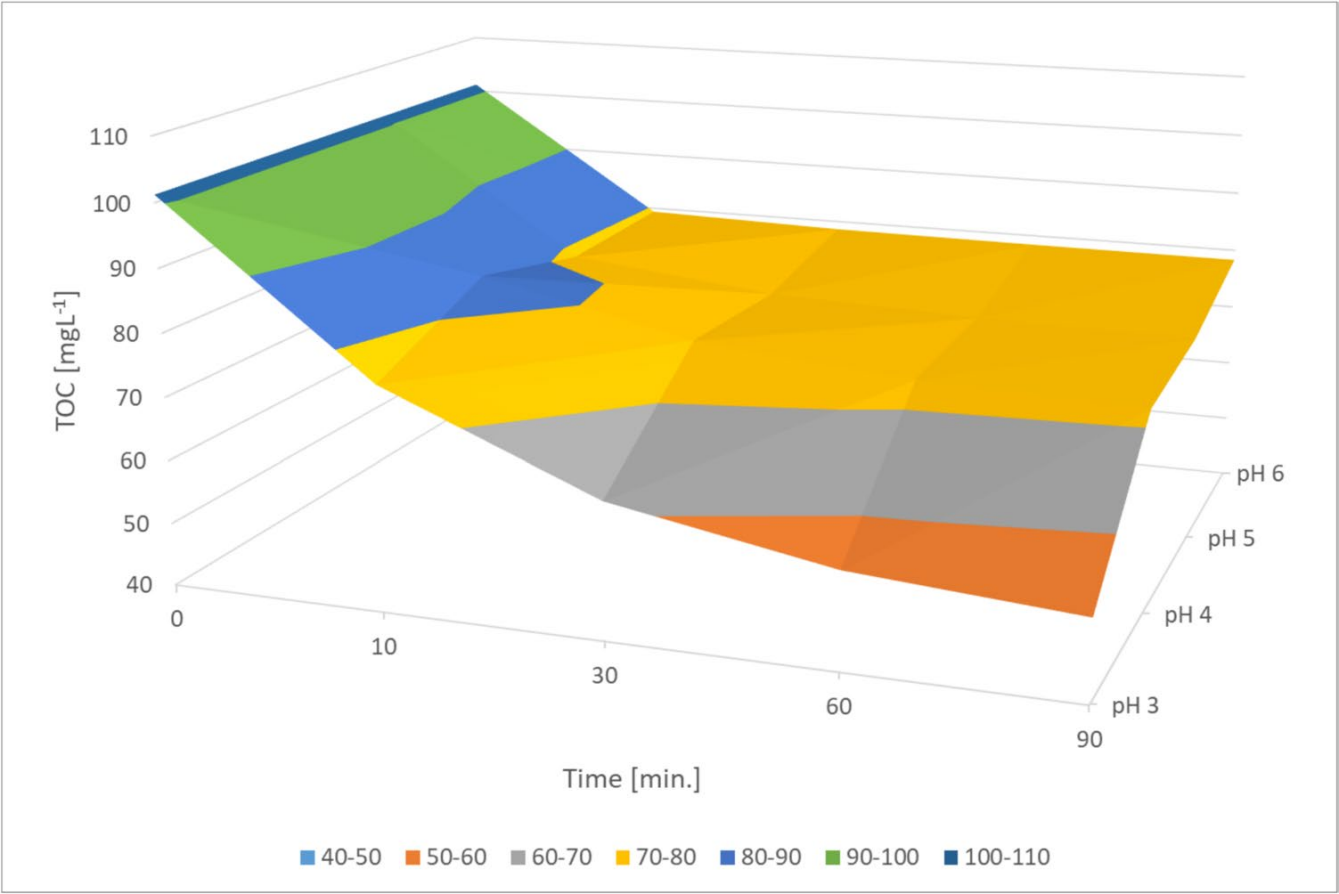
ANOVA calculations results

Variance analysis (ANOVA) was used to examine the variation in mean TOC concentrations over the experiments. The dependent variable in the analysis is the TOC value. The independent variables in the process include the H₂O₂ dose, or sample pH, and the process running time. The analysis provides information on how the contribution of individual parameters and process running affect the TOC value. The set significance level was 0.05. As a result, it was found that the concentrations of TOC showed a significant relationship with the duration of the process, suggesting that time played a crucial role in the treatment efficiency. It is noteworthy that the H_2_O_2_ dose, although significant, was not a decisive factor. In the wastewater treatment process, the reaction of H_2_O_2_ with available Fe^2+^ ions and the time required to regenerate these ions are essential. However, the pH of the solution proved to be the most important factor affecting the process efficiency. Changing the pH had a decisive effect on the degree of wastewater treatment. The relationship between actual results and ANOVA calculations is shown in Fig. 7.

### Waste iron reusability

The potential for multiple waste iron reuse was tested by separating the catalyst after each preset process and placing this separated material in a new wastewater sample, with the same initial parameters (pH, H_2_O_2_ dosage, and process time). The results of the wastewater TOC content confirm the effectiveness of reusing waste iron as an effective catalyst (Table 2).

**Table 2:** Waste iron reuse comparison

	1st use	2nd usage	3rd usage
TOC [mgL^−1^]	52.68	58.16	56.16

Moreover, waste iron is easily separable material, for example with electromagnetic methods. The possibility of the material’s easy separation facilitates its reuse in the process. Nevertheless, due to the increase in iron solubility with each treatment cycle, it is important to monitor the concentration of dissolved iron in the solution. This underscores the need to implement a final coagulation process prior to wastewater discharge to meet standards. Allover, this analysis allowed for the assessment of the catalyst’s durability and effectiveness in successive cycles, which is crucial from the perspective of its practical application.

## Conclusions

This study aligns with the Best Available Techniques (BAT) regulations by meeting the requirement for compound oxidation—replacing the chlorine utilization with the generation of highly reactive radicals—and coagulation, which completes the treatment process.

This study introduces a novel method for treating wet flue gas desulfurization (WFGD) wastewater using waste iron shavings as a heterocatalyst in a modified Fenton process. Based on the findings, the waste iron possesses a distinctive structure, demonstrating high purity, with an iron content exceeding 70%. Moreover, Raman analysis revealed characteristic peaks corresponding to α-Fe_2_O_3_ hexagonal plates, and Fe_3_O_4_ polyhedral particles within the waste iron structure. These findings suggest the presence of hematite and magnetite, which influence the material’s catalytic properties.

The study showed promising performance in removing contaminants from WFGD wastewater with TOC removal efficiency oscillating around 48%. Further, the modified second-order kinetic reaction was found for the best description of the degradation dynamics, and a comprehensive optimization parameters ANOVA clarifies the dominant effect of pH on process performance. Therefore, optimizing the process pH enhanced the generation of reactive species and promoted more effective contaminant degradation.

However, the oxygen presence on the waste iron grains, shown in the waste iron characteristics study, suggests an oxidation process, which may affect the catalyst’s long-term durability and performance. Therefore, a reusability study was conducted, and the catalyst maintained consistent performance over three process cycles, underscoring its durability and reliability.

Since TOC removal efficiency oscillates around 48%, there is a suspicion that the original contaminants are transformed into smaller compounds. Therefore, in future research, this aspect should be further investigated.

## Data Availability

Data and materials that support the findings of this study are available from the corresponding author upon reasonable request.
